# The Swedish RAND-36: psychometric characteristics and reference data from the Mid-Swed Health Survey

**DOI:** 10.1186/s41687-021-00331-z

**Published:** 2021-08-04

**Authors:** Emma Ohlsson-Nevo, Ayako Hiyoshi, Paulina Norén, Margareta Möller, Jan Karlsson

**Affiliations:** 1 School of Health Sciences, Department of Surgery, Örebro University, Örebro, Sweden; 2 University Health Care Research Center, Faculty of Medicine and Health, Örebro University, Örebro, Sweden; 3 Clinical Epidemiology and Biostatistics, School of Medical Sciences, Örebro University, 701 85 Örebro, Sweden

**Keywords:** RAND-36, Reference data, Quality of life, Swedish

## Abstract

**Background:**

This study aims to evaluate data quality, scaling properties, and reliability of the Swedish RAND-36 in a general population sample and to present reference data for the Swedish population.

**Methods:**

Testing of data quality, scaling assumptions and reliability followed methods recommended for the International Quality of Life Assessment Project, previously used for psychometric testing of SF-36 and RAND-36. Data were collected via regular mail for a random stratified sample of the general population in a Swedish county. Weighted means for RAND-36 scores were used and differences by sex, age, education, and occupational groups were tested.

**Results:**

The response rate was 42%, and the sample comprised 3432 persons (45% men, 55% women) with a median age of 56.9 years. The internal consistency reliability was satisfactory, with Cronbach’s alphas > 0.80 for all eight scales. The percentage of missing items was low, ranging between 1.3% and 3.2%. No floor effects (≥15%) were noted, while ceiling effects were observed for physical functioning, role-functioning/physical, pain, role-functioning/emotional, and social functioning. Item–scale correlations were satisfactory (r ≥ 0.40). Correlations among the physical health scales were strong (range 0.58–0.68) as were the correlations among the mental health scales (range 0.58–0.73). Men reported significantly better health-related quality of life (HRQoL) on all scales, although the gender differences were small. Comparisons among age groups showed approximately equal scores among those 20–29, 30–39, and 40–49 years, while significant decreases in physical health were observed in the older age groups. Substantially worse physical health scores were observed in the oldest age group (80+). Significant differences among age groups were noted also for the mental health scales; however, better energy/fatigue and emotional well-being scores were seen in the older age groups, except for the oldest (80+). Those with university education reported significantly better scores on all scales compared to those with mandatory education.

**Conclusions:**

The study suggests that the Swedish version of RAND-36 is an acceptable and reliable instrument for measuring HRQoL in the general population. The study provides reference data that can be used for norm-based comparisons.

**Supplementary Information:**

The online version contains supplementary material available at 10.1186/s41687-021-00331-z.

## Background

The Medical Outcome Study (MOS) Short Form-36 (SF-36) is the most commonly used generic instrument for measuring health-related quality of life (HRQoL). Two versions of the form with identical items and response options have been presented, the SF-36 version 1 (SF-36v1) [1] and the RAND-36 [2]. RAND-36 and its scoring instructions are publicly available on the RAND Corporation’s website and can be used free of charge, while a license and a license fee are required to use the revised SF-36 version 2 (SF-36v2) [3].

Scale scores for RAND-36 and SF-36v1 are identical for six of the eight scales, whereas scoring algorithms for the pain and general health scales are slightly different. However, Hays et al. demonstrated that the correlation between the scales using the two different scoring algorithms was 0.99 in the MOS study, indicating that the difference is negligible [2].

A Swedish version of SF-36v1 was presented in the early 1990s [4–6], while a Swedish translation of RAND-36 has been available since 2013 [7, 8]. The translation of the Swedish RAND-36 differs slightly from the translation of the Swedish SF-36v1, but the translations are assumed to be sufficiently equivalent to allow comparisons between the two versions [8, 9]. A difference between them, however, is that algorithms for calculation of the physical and mental summary scores (PCS and MCS) obtained for the Swedish SF-36v1 [6], are not available for the Swedish RAND-36 [8].

Reference data for the general population are essential to evaluate whether an individual or a group score is above or below the average and are frequently used for norm-based interpretation of HRQoL results in clinical studies [10–12]. Normative population data for SF-36v1 have been collected in a number of countries such as new Zeeland [13], Canada [14], Norway [15], Italy [16], Brazil [17], Tunisia [18], and Greece [19]. Population norms for the Swedish SF-36v1 were collected in 1991–92 [4, 5] and are presented in detail in the Swedish manual and interpretation guide [6]. However, the Swedish SF-36v1 norms have not been updated. Swedish norms for RAND-36 are currently not available.

The aim of the study was to evaluate data quality, scaling properties, and reliability of the Swedish RAND-36 in a general population sample. A second aim was to present RAND-36 norms for the general population in a central region of Sweden.

## Methods

### Design and setting

The Mid-Swed Health Survey [20] was conducted in Region Örebro County, a central region of Sweden with approximately 290,000 inhabitants. The region contains a larger city, several small towns, and rural areas, and about 250,000 people live in a city or small-town area and 40,000 in rural areas.

A random sample, stratified by sex and age, was selected from the general population. An equal part of men and women were randomly selected. From the age group 20–29 and 30–39 years, 800 persons were recruited to each group, as a lower response rate was expected in these age groups. From all other age groups, 340–480 persons were recruited.

The sample size was calculated setting a power at 80% (α = 0.05) to detect a between-group difference of 10 scale points, which can be considered as a medium difference for scale scores ranging between 0 and 100 [12, 21, 22]. The estimation of sample size was based on the RAND-36 subscale (role-functioning/physical) that requires the largest sample to detect a 10-point difference between two groups. In September 2015, 4040 persons received an invitation to participate along with an information letter via regular mail. The survey comprised a 9-page questionnaire including the Swedish RAND-36 and questions about gender, year of birth, occupation, and level of education [20]. The questionnaire was distributed along with a prepaid return envelope via regular mail. After 2 weeks, a thank-you/reminder card was delivered. If the questionnaire was not returned after 5 weeks, a reminding letter and a new questionnaire was sent via regular mail. Because of a low response rate, an additional sample of 4100 persons were invited in March 2016, according to the same stratification principles.

### Classification of subgroups

Age was grouped into 10-year intervals (20–29, 30–39, etc.) with people 80 years and older in one group.

Education was classified into three categories: mandatory (grade 0–9), high school (grade 10–12), and university education (> 12).

The occupation variable included 11 categories: employed, own company, parental leave, student, in labour market program, job seeker, old age pension, activity or sickness compensation, long term sickness, and other. The following subgroups were created from the occupation variable: 1. Employed and self-employed (including employed, own company and parental leave); 2. Unemployed (participants in labour market programs and job seeker) 3. On sick leave (including activity or sickness compensation, and long term sickness). Persons with old age pension and students were not included in the analysis of occupation.

### Rand-36

The RAND-36 consists of 36 items grouped into eight multi-item scales: physical functioning (PF), role-functioning/physical (RP), pain (P), general health (GH), energy/fatigue (EF), social functioning (SF), role-functioning/emotional (RE), and emotional well-being (EW). An additional item asks about health change (HC) in the past year. Scale scores are summed and transformed into scales ranging from 0 (worst possible health state) to 100 (best possible state). A scale score was calculated if at least half of the items in a scale were answered by the respondent (half-scale method) and missing-item values were imputed using a person-specific mean value based on the non-missing items [1]. The half-scale rule is used in scoring of SF-36; however, this criterion is not used in the standard scoring algorithm for RAND-36.

### Psychometric methods

Testing of data quality, scaling assumptions, and reliability followed methods recommended for the International Quality of Life Assessment (IQOLA) Project [23], previously used for psychometric testing of SF-36 and RAND-36 [24]. Psychometric tests were performed in the total sample and in subgroups by gender, age, education, and occupation.

#### Data quality

Completeness of data was evaluated by calculating the percentage of missing data for each of the 36 items. At the scale level, the percentage of computable scale scores was calculated using the half-scale method.

#### Floor and ceiling effects

Floor and ceiling effects were analyzed by calculating the proportion of participants scoring at the lowest and highest possible levels. At the item level, a floor or ceiling effect was considered if at least 50% of the respondents scored at the minimum or maximum level [25]. At the scale level, an effect was indicated if at least 15% of the respondents scored at the lowest or highest level [26].

#### Reliability

Cronbach’s alpha coefficients were computed to estimate the internal consistency reliability of scale scores. A coefficient of at least 0.70 is considered appropriate for group data, although 0.80 is desirable. A coefficient of 0.90 or better is recommended for individual assessment [27].

#### Test of scaling assumptions

Item–scale correlations, that is, the correlation between each item and its own subscale (corrected for overlap), were calculated. A correlation of 0.40 or greater is considered satisfactory [26]. The correlation between items and other subscales was assessed and considered adequate if the correlations were better with the own scale than with other scales. The significance of a difference between two item-scale correlations was determined using the standard error of the correlation matrix (1/√n). The recommended significance criterion of two standard errors was used [26]. Pearson correlation analysis was performed to assess the correlation between item and scales scores.

#### Inter-scale correlations

The correlations (Pearson correlation) among subscales were tested and interpreted as low (< 0.30), medium (0.30–0.49), or strong (0.50) [22] Hypotheses about the magnitude of inter-scale correlations were based on results of the validation of the Swedish SF-36v1 [6]. The strongest correlation was expected between energy/fatigue and emotional well-being, and the weakest between physical functioning and emotional well-being. According to factor analysis, physical functioning, role-functioning/physical, pain, and general health, are strongly related to physical health, while energy/fatigue, social functioning, role-functioning/emotional, and emotional well-being, are strongly associated to mental health. It was hypothesized that the correlations between the four scales that primarily measure physical health would be strong, as well as between the four scales that primarily measure mental health.

#### Test of group differences

Known-groups analysis was performed to test the sensitivity of the scales and ability to capture expected differences between subgroups based on gender, age, education, and occupation [12]. Based on the results of the validation of the Swedish SF-36v1 [5, 6], we assumed: a) that men report better health than women on all eight scales; b) that the four physical health scales gradually deteriorate with age; c) that the differences based on age are smaller for the four mental health scales; d) that those with a low level of education, the unemployed, or those on sick leave, report poorer health.

Weighted mean RAND-36 0–100 scale scores were calculated for the total sample and for subgroups based on gender, age, education, and occupation. Weighted mean T-scores were also calculated to improve comparability across subgroups. T-scores have a mean value of 50 and standard deviation of 10 in the total sample and a T-score above 50 indicates better HRQoL compared to the total norm population. A sampling weight was derived to reflect the demographic distribution of age and gender of the Swedish population in 2015, and non-response. Differences in weighted means between two groups were tested using the nonparametric Somers’ D-test [28], and three or more groups were tested using F-test, with taking account for the sampling design. *P*-values for post hoc pairwise comparisons of the weighted means were adjusted for multiple comparisons using Šidák’s method [29], and adjusted *p*-value 5% or lower was considered to be statistically significant. Linear regression was used to examine whether there was linear or quadratic trend by increasing age. To test a linear trend, the seven-level age variable was used as a continuous variable, and to test quadratic trend, the square of the age variable was added to the model. Survey design and the sampling weight were accounted in linear regression models. SAS 9.4 (SAS Institute, Cary, NC, USA), IBM’s Statistics for Windows Version 22 (IBM, Armonk, NY, USA) and Stata SE Version 15 (StataCorp, College Station, Texas, USA) were used for statistical analysis.

## Results

### Sample characteristics

The overall response rate was 42% and the final sample comprised 3422 participants [20]. The response rate varied according to age and was 61% in participants older than 60 years, 42% in participants 40–59 years, and 28% in the youngest participants (20–39 years old). The sample had a lower proportion of people aged 20–59 and a greater representation of people aged 60 years and older compared with the general Swedish population (Table 1). The weighted sample had the same gender and age distribution as the Swedish population. The age and sex distributions of participants and non-respondents did not differ markedly.

**Table 1:** Demographic characteristics of the study sample and the population in Region Örebro county and Sweden 2015, No (%)

Characteristics	Study sample, Region Örebro county (20+ years)	Weighted sample	Population in Region Örebro county (20+ years) 2015^a^	Population in Sweden (20+ years) 2015^a^
Total	3422	7,611,402	224,801	7,611,402
Gender				
Female	1872 (55)	3,834,333 (50)	113,513 (50)	3,834,333 (50)
Male	1550 (45)	3,777,069 (50)	111,288 (50)	3,777,069 (50)
Age (year)				
Mean	57	50	52	51
Median	60	49	51	50
Range	20–100	20–100	20–100+	20–100+
Age groups				
20–29	394 (12)	1,338,610 (18)	39,697 (18)	1,338,610 (18)
30–39	489 (14)	1,227,641 (16)	33,314 (15)	1,227,641 (16)
40–49	408 (12)	1,314,367 (17)	37,706 (17)	1,314,367 (17)
50–59	399 (12)	1,224,659 (16)	35,426 (16)	1,224,659 (16)
60–69	587 (17)	1,147,578 (15)	35,679 (16)	1,147,578 (15)
70–79	625 (18)	856,646 (11)	27,438 (12)	856,646 (11)
80+	520 (15)	501,650 (7)	15,541 (7)	501,650 (7)
Education				
Mandatory	806 (24)	1,245,098 (17)	55,233 (23)	1,694,024 (21)
High school	1471 (43)	3,605,698 (48)	110,151 (46)	3,449,936 (43)
University	1102 (33)	2,672,718 (36)	68,773 (29)	2,717,115 (34)

^a^Statistics Sweden (https://www.scb.se/en)

A total of 3422 people, 55% women and 45% men, participated in the study. Mean (SD) age was 56.9 (20.1) years (range 20–100 years). Half of the participants were 60 years or older.

Most participants (89%) were born in Sweden. One-third (33%) had university education, 43% had high school education, and 24% had mandatory education (Table 1). Approximately half (46%) of the participants were employed/self-employed, 39% were retirees, 4% were students, 3% on sick leave, and 3% were unemployed.

### Measurement properties

#### Completeness of data

The percentage of missing items was generally low, ranging from 1.3% to 3.2%, averaging 2.2%. The proportion of participants for whom scale scores were computable was consistently high and ranged from 97.1% for role-functioning/emotional to 99% for social functioning (SF) (Table 2). Most subgroups had less than 2% missing scale scores, except for the unemployed with 5.8% missing, the oldest age group (80+) with 4.6% missing, and those with mandatory education with 3.0% missing scores (not presented in a table).

**Table 2:** Descriptive statistic and features of the RAND-36 score distribution (*n* = 3422)

	Physical functioning (PF)	Role-functioning/physical (RP)	Pain (P)	General health (GH)	Energy/fatigue (EF)	Social functioning (SF)	Role-functioning/emotional (SF)	Emotional well-being (SF)
Mean (SD)^a^	78.9 (26.4)	70.4 (40.3)	72.6 (23.4)	66.1 (21.8)	61.0 (22.6)	81.7 (24.2)	74.7 (87.5)	76.2 (19.0)
CI 95%^b^	78.0–79.8	69.1–71.8	71.7–73.5	65.4–66.8	60.2–61.8	80.9–82.5	73.5–76.0	75.5–76-8
Median	90.0	100	77.5	70.0	65.0	100	77.5	80.0
Skewness	−1.345	−.895	−.736	−.554	−.446	−1.270	− 1.079	−.945
Kurtosis	.768	−.931	−.441	−.323	−.379	.793	−.468	.505
Range	1–100	1–100	1–100	1–100	1–100	1–100	1–100	1100
Floor (%)^c^	1.1	18.7	1.4	0.1	0.8	0.8	13.6	0.1
Ceiling (%)^d^	30.3	58.5	28.7	4.8	3.5	51.3	64.2	8.9
Computable scale scores (%)^e^	98.8	97.5	98.8	98.4	98.6	99.0	97.1	98.5

^a^Unweighted means. Higher scores indicate better HRQoL

^b^95% confidence interval

^c^Percentage of subjects with lowest possible score

^d^Percentage of subjects with highest possible score

^e^Percentage computable scale scores

#### Floor and ceiling effects

On the item level, the proportion of subjects who chose the lowest response option (floor effect) varied between 0.8% for emotional well-being item 2 (EW2) and 33.0% for role-functioning/physical item 2 (RP2) (items are presented in Appendix 1). The proportion who chose the highest response option (ceiling effect) varied between 7.5% (EF2) and 70% (EW2). No floor effects (more than 50% at the lowest level) were noted, but ceiling effects (more than 50% at the highest level) were observed for all role-functioning/physical, pain, role-functioning/emotional, and social functioning items, all physical functioning items except PF1, one general health item (GH2), and two emotional well-being items (EW1, EW2).

On the scale level, the proportion who scored at the lowest level varied from 0.1% for emotional well-being and general health to 18.7% for role-functioning/physical (Table 2). The proportion who scored at the highest level varied from 3.5% for energy/fatigue to 64.2% for role-functioning/emotional. Floor effects (more than 15% at the lowest level) were found for role-functioning/physical, while ceiling effects (more than 15% at the highest level) were noted for role-functioning/emotional, role-functioning/physical, social functioning, and pain.

#### Internal consistency reliability

In the total sample, internal consistency reliability coefficients (Cronbach’s alpha) were greater than 0.80 for all scales (Table 3). Alphas above the 0.90 level were noted for physical functioning and role-functioning/physical. In subgroups, the reliability coefficients varied between 0.73 and 0.94 and were above 0.80 in most analyses (93%).

**Table 3:** Internal consistency reliability and range of item-scale correlations (*n* = 3422)

	Reliability		Range of item-scale correlations	
Scale	k^a^	Cronbach’s alpha	Item convergent validity^b^	Item discriminant validity^c^
Physical functioning	10	0.94	0.63–0.85	0.22–0.65
Role-functioning/physical	4	0.91	0.77–0.83	0.35–0.66
Pain	2	0.88	0.81	0.35–0.65
General health	5	0.82	0.50–0.75	0.29–0.66
Energy/fatigue	4	0.85	0.62–0.76	0.31–0.65
Social functioning	2	0.84	0.73	0.44–0.65
Role-functioning/emotional	3	0.84	0.67–0.74	0.31–0.57
Emotional well-being	5	0.84	0.62–0.70	0.21–0.68

^a^Number of items

^b^Correlations between items and hypothesized scale (corrected for overlap)

^c^Correlations between items and other scales This table summarizes data from Appendix 2

#### Test of scaling assumptions

In the total sample, item–scale correlations were satisfactory (r ≥ 0.40) (Appendix 2, Table 3). Significantly higher correlations between items and other scales were supported for all items except two items (EW3, EW5) that correlated highly in both emotional well-being and energy/fatigue (items are presented in Appendix 1).

#### Inter-scale correlations

As expected, the weakest inter-scale correlation was noted between physical functioning and emotional well-being (r = 0.32), while the strongest correlation was seen for energy/fatigue and emotional well-being (r = 0.73) (Appendix 2). Correlations among the physical health scales (PF, RP, P, GH) were strong and ranged between 0.58 and 0.68. Also, correlations among the mental health scales (EF, SF, RE, EW) were strong and varied between 0.58 and 0.73. General health correlated strongly with other scales (range 0.50–0.66) and was most strongly associated with energy/fatigue (r = 0.66). Energy/fatigue showed a medium to strong correlation with other scales (range 0.43–0.73) and was most weakly associated with physical functioning (r = 0.43).

### Swedish RAND-36 scores in the general population

Weighted mean RAND-36 scores were calculated for the total sample as well as for categories by gender, age, education, and occupation, and are presented in Tables 4 and 5. Weighted mean T-scores for the subgroups are presented in supplementary Tables 6 and 7.

**Table 4:** Weighted mean (SD) RAND-36 scale scores by gender and age group

	Physical functioning (PF)	Role functioning/ physical (RP)	Pain (P)	General health (GH)	Energy/fatigue (EF)	Social functioning (SF)	Role functioning/ emotional (RE)	Emotional well-being (EW)
Total	83.5 (23.9)	75.4 (37.6)	74.7 (25.8)	68.1 (21.5)	61.0 (22.2)	82.8 (23.4)	76.7 (36.5)	76.1 (18.6)
Gender								
Men	85.6 (21.5)	77.8 (34.3)	76.3 (23.9)	69.3 (19.4)	63.6 (20.4)	84.8 (21.4)	79.9 (32.8)	78.1 (17.3)
Women	81.4 (26.0)	73.1 (40.8)	73.1 (27.5)	66.9 (23.5)	58.5 (23.7)	80.8 (25.3)	73.4 (39.8)	74.1 (19.8)
*P*-value^a^	< 0.001	0.002	0.001	0.031	< 0.001	< 0.001	< 0.001	< 0.001
Age group								
20–29	91.7 (14.3)^A^	83.3 (25.1)^A^	82.2 (18.1)^A^	71.7 (16.8)^A^	58.9 (16.8)^BC^	83.5 (17.3)^A^	76.6 (29.1)^AB^	73.2 (14.4)^B^
30–39	91.1 (16.5)^A^	81.8 (31.0)^AB^	80.1 (21.9)^AB^	71.5 (19.3)^A^	57.7 (19.4)^BC^	82.9 (21.1)^A^	75.0 (35.0)^B^	74.8 (16.1)^B^
40–49	88.9 (16.3)^AB^	79.6 (28.7)^AB^	76.2 (20.5)^BC^	70.6 (16.8)^AB^	59.4 (17.6)^BC^	83.5 (18.7)^A^	77.6 (29.6)^AB^	75.6 (14.2)^B^
50–59	85.1 (18.7)^B^	77.3 (31.7)^AB^	71.3 (22.9)^CD^	67.0 (19.1)^BC^	61.4 (20.3)^B^	82.1 (21.6)^A^	78.8 (30.7)^AB^	76.6 (17.5)^AB^
60–69	80.3 (24.3)^C^	75.8 (40.4)^B^	71.8 (27.9)^CD^	67.0 (22.8)^BC^	66.6 (23.8)^A^	85.7 (23.6)^A^	82.4 (35.3)^A^	79.5 (20.2)^A^
70–79	72.6 (33.2)^D^	65.2 (53.2)^C^	69.8 (34.3)^D^	64.5 (27.0)^C^	66.8 (28.6)^A^	83.6 (30.1)^A^	77.4 (46.5)^AB^	79.5 (24.3)^A^
80+	49.7 (43.2)^E^	36.4 (64.7)^D^	60.0 (41.8)^E^	53.7 (31.2)^D^	55.4 (35.3)^C^	72.4 (42.6)^B^	57.3 (64.8)^C^	73.3 (30.8)^B^
*F*-value	160.63	72.76	37.56	41.11	20.37	12.27	17.63	10.09
*P*-value^b^	< 0.001	< 0.001	< 0.001	< 0.001	< 0.001	< 0.001	< 0.001	< 0.001
*P*-value for linear trend^c^	< 0.001(q)	< 0.001 (q)	< 0.001	< 0.001 (q)	< 0.001 (q)	0.007 (q)	0.071 (q)	< 0.001 (q)

^a^Comparison of men vs. women (Somers’ D test)

^b^Comparisons of means by age group (F-test)

^c^*p*-value for linear trend was obtained from linear regression, and (q) indicates that there was a statistically significant quadratic trend (*p* < 0.05)

Pairwise comparisons: mean values that share the same capital letter (A-E) do not differ significantly, while groups with different letters have significantly different mean values (Šidák’s test, *p* < 0.05)

**Table 5:** Weighted mean (SD) RAND-36 scale scores by educational level and occupational group

	Physical functioning (PF)	Role functioning/physical (RP)	Pain (P)	General health (GH)	Energy/fatigue (EF)	Social functioning (SF)	Role functioning/ emotional (RE)	Emotional well-being (EW)
	Mean (SD)	Mean (SD)	Mean (SD)	Mean (SD)	Mean (SD)	Mean (SD)	Mean (SD)	Mean (SD)
Educationa								
Mandatory	68.4 (35.1)^A^	59.3 (52.2)^A^	66.2 (34.2)^A^	59.4 (26.6)^A^	60.1 (29.0)^A^	78.5 (31.9)^A^	70.7 (47.4)^A^	75.8 (25.3)^A^
High school	84.4 (22.0)^B^	76.7 (34.9)^B^	73.7 (24.8)^A^	68.8 (20.3)^B^	60.9 (21.2)^B^	83.2 (22.1)^B^	78.4 (33.7)^B^	76.1 (18.0)^A^
University	89.7 (17.4)^C^	81.1 (32.5)^C^	80.1 (21.7)^B^	71.2 (19.6)^B^	61.7 (20.2)^B^	84.2 (21.0)^B^	77.2 (34.7)^AB^	76.3 (16.4)^A^
*F*-value	28.88	10.50	23.09	19.36	5.05	4.91	3.82	1.52
*P*-value^b^	< 0.001	< 0.001	< 0.001	< 0.001	0.007	0.007	0.022	0.219
Occupation^c^								
Employed	90.8 (16.1)^A^	83.2 (31.3)^A^	78.4 (22.9)^A^	71.9 (19.4)^A^	61.7 (20.7)^A^	85.8 (20.3)^A^	81.5 (32.8)^A^	77.9 (16.3)^A^
Unemployed	79.1 (26.4)^B^	72.8 (36.0)^B^	73.2 (28.1)^A^	63.3 (20.0)^B^	55.6 (23.6)^A^	76.3 (26.3)^B^	63.3 (40.9)^B^	66.5 (20.3)^B^
On sick leave	56.3 (30.2)^C^	24.3 (39.8)^C^	42.0 (30.9)^B^	38.1 (24.3)^C^	34.1 (22.7)^B^	48.8 (29.4)^C^	36.9 (42.1)^C^	51.1 (21.8)^C^
*F*-value	70.30	95.90	64.13	87.33	64.11	76.99	56.13	75.83
*P*-value^b^	< 0.001	< 0.001	< 0.001	< 0.001	< 0.001	< 0.001	< 0.001	< 0.001

^a^Level of education: Mandatory (9 years or less); High school (10–12 years); University (> 12 years)

^b^Comparisons of means adjusted for age (F-test)

Pairwise comparisons; age-adjusted mean values that share the same capital letter (A, B, and C) do not differ significantly, while categories with different letters have significantly different mean values (Šidák’s test, *p < 0.05*)

^c^Occupation categories: Employees and self-employed; Unemployed (job seeker and participants in labor market programs); On sick leave (activity or sickness compensation, long term sickness)

#### Sex

Men had significantly better scores then women on all RAND-36 scales, although the differences were small (2.4–6.5 scale points) (Table 4).

#### Age groups

There was statistically significant differences in all RAND-36 scales by age group (Table 4). Post-hoc pairwise comparisons between the age groups 20–29, 30–39 and 40–49 showed that there was only one significant difference, i.e. pain was significantly worse at the ages of 40–49 years compared with those aged 20–29 years. As expected, decreases in physical health (PF, RP, P and GH) were observed with increasing age and substantially worse scores were seen in the oldest age groups (70–79 and 80+ years) compared to those aged 20–49 years. The decline by age was most prominent for physical functioning and role-functioning/physical.

Significant differences between age groups were noted also for the mental health scales (EF, SF, RE, and EW); however, better energy/fatigue and emotional well-being scores were seen in the older age groups, except for the oldest group (80+). People between 60 and 79 years reported significantly better energy/fatigue compared to all other age groups, including the younger adults (20–39 years). Energy/fatigue was also significantly better in the 50–59 age group compared to the oldest group (80+). Social functioning scores were roughly equal among those between 20 and 79 years, while significantly worse scores were observed in the oldest group (80+). No significant differences in role-functioning/emotional scores were observed among those aged 20–59 and 70–79 years, but significantly better scores were noted for those aged 20–79 years compared with the oldest (80+) and for those between 60 and 69 years compared with the group 30–39 years. Emotional well-being scores were significantly better in those 60–79 years compared to the younger and middle-aged (20–49 years) and the oldest (80+).

#### Education

Participants with mandatory education as their highest education were older than those with high school or university education, 73.4 (SD 13.7) vs. 48.6 (SD 18.3) and 49.6 (SD 17.8) years, respectively (*p* < 0.0001). Thus, tests of significant differences in HRQoL between education levels were adjusted for age. All age-adjusted RAND-36 scales differed significantly by level of education (Table 5). Pairwise comparisons showed that those with university and high school education reported significantly better scores on five of the RAND-36 scales (physical functioning, role-functioning/physical, general health, energy/fatigue, and social functioning) compared to those with mandatory education. Scores on five scales (general health, energy/fatigue, social functioning, role-functioning/emotional, and emotional well-being were roughly equal among those with high school and university education, whereas three scales (physical functioning, role-functioning/physical, and pain were significantly better in those with university education.

#### Occupation

Participants on sick leave were somewhat older than the employed and unemployed, 49.2 (SD 12.8) vs. 45.4 (SD 13.5) and 43.3 (SD 16.3) years, respectively (*p* = 0.008), and comparisons between groups were adjusted for age. All age-adjusted RAND-36 scales differed significantly by occupation group. Pairwise comparisons showed there were clear differences for all eight RAND-36 scales between employees/self-employed, unemployed, and those on sick leave (Table 5). Employees reported the best health status, while people on sick leave reported the worst health status.

## Discussion

The purpose of the study was to evaluate the psychometric properties of the Swedish version of RAND-36 in the general population and to present Swedish reference values. The performance of the instrument was tested in the total sample as well as in subgroups by gender, age, education, and occupation. The completeness of data was satisfactory both at the item level and at the scale level, indicating that the questionnaire was well accepted by the respondents.

No floor effects (≥15%) for scale scores were observed in the total sample, while ceiling effects were noted, especially for role-functioning/physical, role-functioning/emotional, and social functioning (> 50%) but also for pain and physical functioning (28%–30%). Ceiling effects for these scales are expected in population studies [4, 23]. However, ceiling effects for role-functioning/physical and role-functioning/emotional can also be attributed to the use of dichotomous response options that limit the ability to discriminate between individuals. Also, the social functioning scale consists of only two items with similar wordings, which contributes to ceiling effects and weak discriminatory capacity. Ceiling effects in the total sample were lower for all scales compared to the validation of the Swedish SF-36v1 in the 1990s [4]. One likely explanation is that the response rate in the current study is lower, especially in the younger ages, which leads to a larger proportion of older people with worse scores on the physical health scales.

Satisfactory item–scale correlations (r ≥ 0.40) were confirmed for all eight scales and item–other scale correlations were in most cases acceptable. However, two emotional well-being items correlated equally strongly in the emotional well-being and energy/fatigue domains. Inter-scale correlations also showed that emotional well-being and energy/fatigue were strongly associated (r = 0.73). Cronbach’s alpha for the eight scales in the total sample ranged from 0.82 to 0.94, showing satisfactory internal consistency reliability for group comparisons. Alpha values were roughly at the same levels as in the validation of the Swedish SF-36v1 [4].

Men scored slightly better than women on all RAND-36 scales, in line the previous Swedish SF-36v1 validation study [4] and several other population studies in different countries [15, 17–19, 30]. In the present study, effect size estimates (Cohen’s *d*) indicated trivial gender differences for six scales (physical functioning, role-functioning/physical, pain, general health, social functioning, and role-functioning/emotional, while the differences for energy/fatigue and emotional well-being were in the small range.

As expected, comparisons among age groups showed worse physical health scores in the oldest age groups, especially for physical functioning and role-functioning/physical, which is consistent with results in other normative population studies [5, 13, 15, 17–19, 31]. Analyses of the mental health scales, however, showed better energy/fatigue and emotional well-being scores for those 60–79 years old compared to the youngest, middle-aged, and oldest (80+ years). The latter finding differs from the results in the validation of the Swedish SF-36v1 [5]. In the present study, mean scores on social functioning and role-functioning/emotional were roughly equal between age groups, except for the oldest who reported worse levels. The results regarding the mental health scales in different age groups differ between countries. Some studies have reported better mental health in older persons [15, 31], while the opposite trend has been observed in other countries [5, 18, 19]. Approximately equal mental health scores among age groups have also been reported [17].

As expected, those with university education reported better RAND-36 scores than those with mandatory education. Also, employed persons scored better than those who were on sick leave and the unemployed. These findings are in line with the previous Swedish SF-36v1 validation study [5] as well as most other normative population studies in other countries [15, 18, 19, 31] .

This study provides reference data for RAND-36 collected in 2015–16, based on a random sample of 3422 participants aged 20–100 years from the general population in a region in central Sweden. The demographic structure of the region is similar to many other regions in Sweden, and the data may be suitable as a reference for the general Swedish population. The Swedish SF-36v1 population norms, which were collected in the early 1990s, have frequently been used to compare and interpret the health profiles of patient populations. However, the demographic and socioeconomic changes as well as lifestyle changes in recent decades may well have affected the health status of the population, and it is unclear whether the SF-36v1 reference data are still valid for norm-based evaluations. Comparisons of SF-36 scores between population surveys in 1996, 2002, and 2015 in Norway indicated relatively stable scores over the 19-year period, but significant changes were observed in specific age groups [15].

Comparison of the RAND-36 and SF-36v1 health profiles in the total samples of the present and earlier Swedish validation study show generally worse scores in the current study on all scales except pain. Most scales (role-functioning/physical, general health, energy/fatigue, social functioning and role-functioning/emotional are clearly worse (5.8–9.0 points), while physical functioning and emotional well-being scores are slightly worse (4.4–4.8 points). These differences may to some extent be due to differences in the age distribution in the two studies. The unweighted mean age in the current study was 56.9 years, which is markedly higher than in the SF-36v1 validation study, where the mean age was 42.7 years [4]. However, in the present study a sampling weight was used to accurately reflect the demographic distribution of the Swedish population in 2015. The weighted mean age was 50 years, which is equal to the whole Swedish population but still significantly higher than in the previous validation study of SF-36v1.

The study presents no summary measures for physical and mental health, as there is no standard procedure available for the RAND-36. It should be noted that the SF-36 method for scoring the physical and mental component scores has been criticized, since the calculations in some cases tend to produce scores that deviate from the results obtained for the corresponding subscales [32, 33]. However, alternative scoring algorithms for the RAND-36 summary measures that differ from the SF-36 standard method have been proposed [33].

The large sample size (*n* = 3422) is a strength of the study that made it possible to repeat the psychometric analyses in several subgroups. According to recommendations in the IQOLA project, the sample size for norming of SF-36v1 in the general population should be approximately 2500 to 3000 participants [23]. The low response rate (42%) is a weakness that may affect the representativeness of the respondents and introduce bias in examined associations, even though this is not necessarily so [34]. The response rate was higher among those 60 years of age and older (61%), while only 28% in the youngest age groups (20–39 years) responded. The response rate in the present study is considerably lower than in the previous validation study of the SF-36v1 with a response rate of 68% [4]. The response rate has declined, especially in the younger ages, in several Swedish general population surveys during the last decades [35]. For example, the response rates in the Swedish national public health surveys have gradually decreased from 60% in 2004 to 47% in 2016. The current study was distributed by regular mail, but it may be advisable to include internet-based assessment to improve the response rate. To compensate for the expected low response rate in the younger age groups, the sample was stratified by age to obtain a sufficient number of respondents in all age groups. We also used a sampling weight to adjust the age and gender distribution to the general Swedish population, which means that the normative RAND-36 data presented in the study are estimated to be comparable to the Swedish population in 2015.

## Conclusion

The study suggests that the Swedish version of RAND-36 is an acceptable and reliable instrument for measuring HRQoL in the general population. The study provides reference data that can be used for norm-based comparisons.

## Supplementary Information

**Additional file 1: Table 6.** Weighted mean (SD) T-scores for the RAND-36 scales by gender and age group.

**Additional file 2: Table 7.** Weighted mean (SD) T-scores for the RAND-36 scales by educational level and occupational group.

**Additional file 3: Appendix 1.** Item description.

**Additional file 4: Appendix 2.** RAND-36 item descriptive statistics, inter-scale, item-total (corrected for overlap), and item-other scale correlations (*n* = 3422).

## Data Availability

The data that support the findings of this study are available from University Health Care Research Center, Faculty of Medicine and Health, Örebro University, Örebro, Sweden, but restrictions apply to the availability of these data, which were used under license for the current study, and so are not publicly available.

## Abbreviations

ANCOVA: One-way analysis of covariance
EF: Energy/fatigue
EW: Emotional well-being
GH: General Health
HRQoL: Health-related quality of life
IQOLA: International Quality of Life Assessment
P: Pain
PF: Physical functioning
RP: Role-functioning/physical
SF-36: The Medical Outcome Study Short Form-36
SF: Social functioning
RE: Role-functioning/emotional
