# Supplementary material for: The Swedish RAND-36: psychometric characteristics and reference data from the Mid-Swed Health Survey
Source: J Patient Rep Outcomes. 2021 Aug 4;5:66. doi: 10.1186/s41687-021-00331-z (PMC8339183; doi:10.1186/s41687-021-00331-z)
Supplement: Supplementary file 1 — Additional file 1: Table 6. Weighted mean (SD) T-scores for the RAND-36 scales by gender and age group. [file 41687_2021_331_MOESM1_ESM.pdf]

Table 6. Weighted mean (SD) T-scores for the RAND-36 scales by gender and age group.

	Physical functioning (PF)	Role functioning/ physical (RP)	Pain (P)	General health (GH)	Energy/fatigue (EF)	Social functioning (SF)	Role functioning/ emotional (RE)	Emotional well-being (EW)
Total	50.0 (10.0)	50.0 (10.0)	50.0 (10.0)	50.0 (10.0)	50.0 (10.0)	50.0 (10.0)	50.0 (10.0)	50.0 (10.0)
Gender								
Men	50.9 (9.0)	50.6 (9.1)	50.6 (9.3)	50.6 (9.0)	51.2 (9.2)	50.9 (9.1)	50.9 (9.0)	51.1 (9.3)
Women	49.1 (10.9)	49.4 (10.8)	49.4 (10.7)	49.4 (10.9)	48.9 (10.7)	49.2 (10.8)	49.1 (10.9)	48.9 (10.6)
Age group								
20-29	53.4 (6.0)	52.1 (6.7)	52.9 (7.0)	51.7 (7.8)	49.1 (7.6)	50.3 (7.4)	50.0 (8.0)	48.4 (7.7)
30-39	53.2 (6.9)	51.7 (8.2)	52.1 (8.5)	51.6 (9.0)	48.5 (8.7)	50.0 (9.0)	49.5 (9.6)	49.3 (8.6)
40-49	52.3 (6.8)	51.1 (7.6)	50.6 (8.0)	51.2 (7.8)	49.3 (7.9)	50.3 (8.0)	50.3 (8.1)	49.7 (7.6)
50-59	50.7 (7.8)	50.5 (8.4)	48.7 (8.9)	49.5 (8.9)	50.2 (9.1)	49.7 (9.2)	50.6 (8.4)	50.3 (9.4)
60-69	48.7 (10.2)	50.1 (10.7)	48.9 (10.8)	49.5 (10.6)	52.5 (10.7)	51.2 (10.1)	51.6 (9.7)	51.8 (10.8)
70-79	45.4 (13.9)	47.3 (14.1)	48.1 (13.3)	48.3 (12.6)	52.6 (12.9)	50.3 (12.9)	50.2 (12.8)	51.8 (13.1)
80+	35.8 (18.1)	39.6 (17.2)	44.3 (16.2)	43.3 (14.5)	47.5 (15.9)	45.6 (18.2)	44.7 (17.8)	48.5 (16.5)
