# Supplementary material for: The Swedish RAND-36: psychometric characteristics and reference data from the Mid-Swed Health Survey
Source: J Patient Rep Outcomes. 2021 Aug 4;5:66. doi: 10.1186/s41687-021-00331-z (PMC8339183; doi:10.1186/s41687-021-00331-z)
Supplement: Supplementary file 2 — Additional file 2: Table 7. Weighted mean (SD) T-scores for the RAND-36 scales by educational level and occupational group. [file 41687_2021_331_MOESM2_ESM.pdf]

Table 7. Weighted mean (SD) T-scores for the RAND-36 scales by educational level and occupational group.

	Physical functioning (PF)	Role functioning/ physical (RP)	Pain (P)	General health (GH)	Energy/fatigue (EF)	Social functioning (SF)	Role functioning/ emotional (RE)	Emotional well-being (EW)
Education[a]								
Mandatory	43.7 (14.7)	45.7 (13.9)	46.7 (13.3)	46.0 (12.4)	49.6 (13.1)	48.2 (13.6)	48.4 (13.0)	49.9 (13.6)
High school	50.4 (9.2)	50.3 (9.3)	49.6 (9.6)	50.3 (9.4)	50.0 (9.6)	50.2 (9.5)	50.5 (9.3)	50.0 (9.6)
University	52.6 (7.3)	51.5 (8.6)	52.1 (8.4)	51.5 (9.1)	50.3 (9.1)	50.6 (9.0)	50.1 (9.5)	50.1 (8.8)
Occupation[b]								
Employed	53.1 (6.7)	52.1 (8.3)	51.4 (8.9)	51.8 (9.0)	50.3 (9.3)	51.3 (8.7)	51.3 (9.0)	51.0 (8.8)
Unemployed	48.2 (11.1)	49.3 (9.6)	49.4 (10.9)	47.8 (9.3)	47.6 (10.6)	47.3 (11.2)	46.3 (11.2)	44.9 (10.9)
Sickness	38.6 (12.7)	36.4 (10.6)	37.3 (12.0)	36.1 (11.3)	37.9 (10.2)	35.5 (12.5)	39.1 (11.5)	36.6 (11.7)

[a]Level of education: Mandatory (9 years or less); High school (10-12 years); University (>12 years).

[b]Occupation categories: Employees and self-employed; Unemployed (job seeker and participants in labor market programs); On sick leave (activity or sickness compensation, long term sickness).
