# Supplementary material for: The Swedish RAND-36: psychometric characteristics and reference data from the Mid-Swed Health Survey
Source: J Patient Rep Outcomes. 2021 Aug 4;5:66. doi: 10.1186/s41687-021-00331-z (PMC8339183; doi:10.1186/s41687-021-00331-z)
Supplement: Supplementary file 3 — Additional file 3: Appendix 1. Item description. [file 41687_2021_331_MOESM3_ESM.pdf]

## Appendix 1. Item description

*Physical functioning (PF)*

PF1. Vigorous activities

*Role-functioning/physical (RP)*

RP2. Accomplished less than would like

*General health (GH)*

GH2. My health is excellent

*Energy/fatigue (EF)*

EF2. Have a lot of energy

*Emotional well-being (EW)*

EW1. Been a very nervous person

EW2. Felt so down in the dumps nothing could cheer you up

EW3. Felt calm and peaceful

EW5. Been a happy person
