# Supplementary material for: The Swedish RAND-36: psychometric characteristics and reference data from the Mid-Swed Health Survey
Source: J Patient Rep Outcomes. 2021 Aug 4;5:66. doi: 10.1186/s41687-021-00331-z (PMC8339183; doi:10.1186/s41687-021-00331-z)
Supplement: Supplementary file 4 — Additional file 4: Appendix 2. RAND-36 item descriptive statistics, inter-scale, item-total (corrected for overlap), and item-other scale correlations (n = 3422). [file 41687_2021_331_MOESM4_ESM.pdf]

Appendix 2. RAND-36 item descriptive statistics, inter-scale, item-total (corrected for overlap), and item-other scale correlations (n=3422).

**Scale, item**	**Mean**	**SD**	**% missing**	**PF**	**RP**	**P**	**GH**	**EF**	**SF**	**RE**	**EW**
Physical functioning (PF)*				**-**	***.68***	***.59***	***.58***	***.43***	***.50***	***.39***	***.32***
1	2.07	0.80	2.0	**.63**	.55	.51	.50	.32	.34	.27	.22
2	2.59	0.65	1.5	**.85**	.65	.55	.54	.42	.47	.37	.33
3	2.63	0.63	2.5	**.79**	.58	.52	.49	.38	.44	.34	.29
4	2.52	0.70	1.5	**.84**	.60	.51	.51	.37	.41	.33	.27
5	2.74	0.54	2.2	**.80**	.53	.44	.45	.34	.41	.32	.27
6	2.46	0.72	2.6	**.74**	.52	.49	.46	.32	.35	.29	.22
7	2.51	0.72	1.9	**.84**	.61	.51	.51	.37	.42	.32	.27
8	2.68	0.63	2.5	**.84**	.57	.47	.45	.34	.41	.32	.26
9	2.80	0.51	2.7	**.75**	.46	.40	.38	.29	.40	.30	.25
10	2.83	0.46	1.4	**.64**	.43	.40	.38	.32	.43	.31	.30
Role-functioning/physical (RP)*				***.68***	-	***.63***	***.61***	***.54***	***.59***	***.56***	***.41***
1	1.77	0.42	3.0	.58	**.77**	.52	.51	.46	.53	.51	.36
2	1.66	0.47	2.5	.55	**.77**	.52	.55	.52	.54	.54	.39
3	1.69	0.46	3.1	.62	**.83**	.58	.55	.47	.51	.47	.36
4	1.70	0.59	3.0	.66	**.81**	.59	.55	.47	.52	.47	.35
Pain (P)*				***.59***	***.63***	-	***.61***	***.56***	***.57***	***.41***	***.43***
1	4.40	1.39	1.5	.52	.54	**.81**	.56	.51	.49	.35	.38
2	4.09	1.09	2.0	.61	.65	**.81**	.60	.55	.59	.43	.43
General health (GH)*				***.58***	***.61***	***.61***	-	***.66***	***.61***	***.50***	***.56***
1	3.27	1.06	1.5	.60	.61	.59	**.69**	.60	.56	.48	.49
2	4.28	1.04	1.6	.31	.36	.36	**.52**	.40	.40	.32	.36
3	3.74	1.23	2.0	.40	.44	.41	**.58**	.46	.43	.33	.40
4	3.45	1.15	1.9	.37	.36	.37	**.50**	.40	.35	.29	.33
5	3.48	1.25	1.6	.53	.56	.58	**.75**	.66	.59	.47	.54
Energy/fatigue (EF)*				***.43***	***.54***	***.56***	***.66***	-	***.67***	***.58***	***.73***
1	4.05	1.29	1.6	.39	.49	.50	.61	**.76**	.60	.51	.65
2	3.55	1.42	2.9	.37	.47	.44	.56	**.64**	.53	.48	.61
3	4.61	1.37	2.5	.31	.39	.46	.48	**.62**	.53	.44	.57
4	3.98	1.36	1.5	.35	.45	.46	.54	**.75**	.57	.48	.60
Social functioning (SF)*				***.50***	***.59***	***.57***	***.61***	***.67***	-	***.64***	***.66***
1	4.29	1.04	1.7	.48	.57	.55	.57	.61	**.73**	.61	.60
2	4.25	1.03	2.4	.44	.53	.50	.57	.65	**.73**	.58	.64
Role-functioning/emotional (RE)*				***.39***	***.56***	***.41***	***.50***	***.58***	***.64***	-	***.61***
1	1.78	0.42	3.1	.38	.53	.37	.45	.49	.57	**.71**	.53
2	1.67	0.47	2.6	.33	.50	.36	.44	.53	.56	**.74**	.55
3	1.80	0.40	3.2	.31	.43	.34	.40	.48	.55	**.67**	.51
Emotional well-being (EW)*				***.32***	***.41***	***.43***	***.56***	***.73***	***.66***	***.61***	-
1	5.24	1.07	1.7	.21	.27	.27	.36	.47	.47	.45	**.62**
2	5.49	0.97	2.1	.28	.31	.33	.41	.51	.55	.49	**.67**
3	4.20	1.42	2.1	.25	.37	.38	.50	.68	.56	.50	**.68**
4	5.06	1.17	2.2	.23	.31	.32	.43	.59	.54	.52	**.70**
5	4.05	1.42	2.1	.31	.36	.37	.49	.60	.51	.46	**.62**
Health change (HC)*	2.97	0.79	1.3	***.34***	***.35***	***.35***	***.38***	***.33***	***.34***	***.26***	***.26***

*Inter-scale correlations. Two standard error of the correlation matrix = 0.034.
